# Various types of mycorrhizal fungi sequences detected in single intracellular vesicles

**DOI:** 10.5511/plantbiotechnology.25.0228a

**Published:** 2025-09-25

**Authors:** Enkhtugs Erdenetugs, Shunsuke Harada, Enkhmaa Erdenetugs, Takeshi Sentoku, Michio Arai, Katsuharu Saito, Yoshihiro Kobae

**Affiliations:** 1 Department of Sustainable Agriculture, Rakuno Gakuen University, 582 Bunkyodai Midori-machi, Ebetsu, Hokkaido 069-0836, Japan; 2 Faculty of Agriculture, Shinshu University, Minamiminowa, Nagano 399-4598, Japan; 3 Arai Helmet Ltd., 2-12 Azuma-cho, Omiya-ku, Saitama-shi, Saitama 330-0841, Japan

**Keywords:** arbuscular mycorrhizal fungi, fine root endophytes, genetic heterogeneity, intracellular vesicle, Mucoromycota

## Abstract

A diverse range of microbes have been observed to coexist in plant roots in the field, among which arbuscular mycorrhizal fungi (AMFs) are universal and have recently been shown to be of two types: one belonging to the subphylum Glomeromycotina (G-AMF) and the other to the subphylum Mucoromycotina (M-AMF). These two types of mycorrhizal fungi are known to co-occur in roots. This is because, in addition to the morphological evidence, diverse ribosomal RNA (rRNA) gene sequences, including those of G-AMF, are detected in mycorrhizae colonized with M-AMF. However, it is difficult to physically distinguish between these AMFs, and amplification bias of G-AMF and M-AMF by PCR has hampered analysis of the detailed symbiotic behaviour of both AMFs. In this study, we isolated a single vesicle of lipid-accumulating AMF in the root and sequenced its rRNA gene by PCR using uniquely designed primers with reduced amplification bias. Notably, G-AMF and M-AMF rRNA gene sequences were detected in one vesicle. These results suggest new avenues for mycorrhizal research on the overlooked morphology of AMF vesicles and their mode of genetic co-occurrence of G-AMF and M-AMF.

## Introduction

The lifestyle and functionality of arbuscular mycorrhizal fungi (AMF) belonging to the subphylum Glomeromycotina (G-AMF) in agroecosystems and natural ecosystems has been well studied. G-AMF form nutrient exchange organs in host plants, called arbuscules, within the root cortical cells. The host plants provide sugars and lipids to G-AMF (Rich et al. 2017), while the G-AMF provides inorganic nutrients such as phosphate and ammonium to the plant (Hodge and Storer 2015; Karandashov and Bucher 2005). In agricultural fields and natural soils, nutrient exchange between G-AMF and plants plays an important role in nutrient cycling and the provision of ecosystem services (Douds and Millner 1999; Powell and Rillig 2018).

In addition to G-AMF, Mucoromytotina AMF (M-AMF) have recently attracted attention (Prout et al. 2024). They have long been known as fine root endophytes (FRE) (Walker et al. 2018), with thin mycelia and characteristic morphology, such as intercalary vesicles in the cortical layer, that can be distinguished from G-AMF colonization using high-magnification microscopy (Orchard et al. 2017a, b; Thippayarugs et al. 1999). Some fungi in the order Endogonales are M-AMF and, like G-AMF, form arbuscules in root cortex cells (Albornoz et al. 2020). Endogonales was originally named *Glomus tenue*, which belongs to G-AMF, but rRNA sequence analysis has shown that Endogonales is now phylogenetically distinct from G-AMF (Walker et al. 2018).

M-AMF has been documented less frequently than G-AMF. Mycorrhizae produced by M-AMF have been reported in agroecosystems and natural ecosystems across a broad range of environments, with the exception of tropical regions (Albornoz et al. 2021). A notable characteristic is the observation that M-AMF is frequently co-occurring with G-AMF in the roots (Field et al. 2019). The separation of these AMFs morphologically and physically in the root is challenging, and there has been only limited success in isolating and culturing M-AMF from G-AMF. Obtaining and analyzing mycorrhizae in which M-AMF is dominant over G-AMF is also very limited (Kowal et al. 2020), and the focus of research on M-AMF has been hindered by its G-AMF background (Orchard et al. 2017b). Nonetheless, recent findings by Katie Field’s research group, establishing a monoxenic culture line of M-AMF, employing tracer experiments with stable isotope nitrogen, have demonstrated that M-AMF receives photosynthetic products from plants and, in return, provides nitrogen to plants (Howard et al. 2024; Hoysted et al. 2023). Information on the rRNA gene sequence and genome structure of M-AMF is also gradually increasing (Cole et al. 2024).

For M-AMF diversity analysis, NS1/EF3, a universal primer set targeting fungal 18S rRNA genes, is often used (Smit et al. 1999; White et al. 1990). However, this primer set amplifies fungi other than mycorrhizal types, as well as plants, and is therefore not suitable for efficient studies targeting trace amounts of M-AMF in roots. EndAD1f/EndAD2r (Desirò et al. 2013) is a primer set that specifically amplifies Endogonales, and is used for the ecological and phylogenetic analysis of this lineage (Desirò et al. 2017; Ogura-Tsujita et al. 2019; Yamamoto et al. 2019). However, these sequences are not found in the genomic sequences of *Sphaerocreas pubescens* (Hirose et al. 2014), which belongs to Endogonomycetes, and is likely to be unable to amplify some species of symbiotic Mucoromytotina. Very recently, Seeliger et al. (2024) modified the PCR primer set AMV4.5NF/AMDGR (Sato et al. 2005), which amplifies a 220 bp region of AMF 18S rRNA gene, to simultaneously amplify G-AMF and M-AMF, including Mortierellomycotina. Some species of Mortierellomycotina have beneficial interactions with plants as root endophytes (Bonfante and Venice 2020; Vandepol et al. 2020; Zhang et al. 2020).

In this study, we independently designed PCR primers that could amplify G-AMF and M-AMF simultaneously. Through a survey of a number of agricultural fields in Japan, we identified fields where M-AMF is commonly found, and in the roots of plants growing in this field, arbuscules of M-AMF were formed in the cortical cells, and at the same time, many lipid droplet-accumulating vesicles were formed within the cells. The vesicles were isolated with a sharpened needle tip and analyzed for their rRNA gene sequences, and surprisingly, both M-AMF and G-AMF sequences were detected in one vesicle. The contamination of mycorrhizal spores with other fungi has long been known (Redecker et al. 1999). It is likely that rRNA genes from several fungal species co-occur within the M-AMF-like vesicles.

## Materials and methods

### Primer design

Nucleotide sequences of 18S rRNA genes from Mucoromycota, Ascomycota, Basidiomycota, and plants were retrieved from the non-redundant SILVA reference dataset (https://www.arb-silva.de (Accessed Apr 1, 2020)) and EUKARYOME v.1.9 (Tedersoo et al. 2024; https://eukaryome.org/ (Accessed Aug 1, 2024)). The sequences were aligned using MAFFT v7.475 (Katoh and Standley 2013). To design primers that specifically targeting the 18S rRNA gene of Mucoromycota fungi colonizing plant roots, we searched for regions that enable primers binding to the genes of most Mucoromycota while excluding Mucoromycetes and avoiding amplification of plant 18S rRNA genes. To enhance specificity, mismatched bases between the primers and plant sequences were replaced with LNA, a nucleic acid analogue with a bicyclic 2′-*O*, 4′-*C*-methylene linked furanose sugar (Egli et al. 2001; Latorra et al. 2003). Finally, we designed a primer set, BM0639-5′-LNA/BM0853-3′-LNA, which amplifies a product of approximately 260 bp spanning the V4 region of the 18S rRNA gene (Table 1).

**Table 1:** Table 1. Primers used in this study.

Primer set	Forward primer (5′ ->3′)	Reverse primer (5′ ->3′)	Annealing temp. (°C)	Note
Basal Mucoromycota-specific primer set				
BM0639-5′-LNA/BM0853-3′-LNA	GTTAAAAAGCTCGTAGTTGAA**T**TT	CAACT**A**TCCCTATTMATCATTAC	60	
with Illumina MiSeq adaptors	ACACTCTTTCCCTACACGACGCTCTTCCGATCTGTTAAAAAGCTCGTAGTTGAA**T**TT	GTGACTGGAGTTCAGACGTGTGCTCTTCCGATCTCAACT**A**TCCCTATTMATCATTAC	60	
Universal primer sets				
Fun18S1-LNA^1^/EF3^2^	CCATGCATGT**C**TAAGTAT**A**A	TCCTCTAAATGACCAAGTTTG	55	for fungi
NS1^3^/EF3	GTAGTCATATGCTTGTCTC	TCCTCTAAATGACCAAGTTTG	55	for *Ustilago maydis*
NS1/EF3-plant^4^	GTAGTCATATGCTTGTCTC	TCCTCTAAATGATAAGGTTCA	55	for plants

^1^Fun18S1 designed by Lord et al. (2002) was modified. ^2^Smit et al. 1999. ^3^White et al. 1990. ^4^EF3 was modified. Locked nucleic acid (LNA) bases are indicated with bold red letters. Illumina MiSeq adaptors are underlined.

### Primer specificity

To test the specificity of the newly designed primers, 18S rRNA genes from diverse fungal and plant species were cloned. Genomic DNA was isolated from fungal and plant materials listed in Table 2 by CTAB method (Tisserant et al. 2013). Partial sequences of fungal and plant 18S rRNA genes were amplified using universal primers NS1/EF3 and NS1/EF3-plant, respectively (Table 1). The PCR products were cloned into the EcoRV site of the plasmid pZErO-2 (Thermo Fisher Scientific). To confirm the primer specificity, PCR mixtures (10 µl) containing KOD One PCR Master Mix (Toyobo), 0.3 µM of each primer (Table 1), and 1 pg µl^−1^ plasmid DNA were prepared. To further test the exclusion of plant 18S rRNA gene amplification even in the presence of excess plant gene copies, plasmid DNA at concentrations of 1, 10, and 100 pg µl^−1^ was added to the PCR mixtures. The following thermal profile was used: 98°C for 10 s, 55 or 60°C for 5 s, 68°C for 5 s, repeated for 35 cycles. The amplified products were separated by electrophoresis on 1.5% agarose gels and visualized with a UV transilluminator after staining with ethidium bromide.

**Table 2:** Table 2. Fungal and plant materials used in this study.

Phylum	Class	Order	Family	Species
Mucoromycota	Glomeromycetes	Glomerales	Glomeraceae	*Rhizoglomus venetianum* DAOM 197198 (synonym *Rhizophagus irregularis*)
Mucoromycota	Mortierellomycetes	Mortierellales	Mortierellaceae	*Linnemannia elongata* MAFF425198 (synonym *Mortierella elongata*)
Mucoromycota	Endogonomycetes	Endogonales	Densosporaceae	*Sphaeroceas pubescens* S7-3
Mucoromycota	Endogonomycetes	Endogonales	Endogonaceae	*Endogone pisiformis* C-3
Mucoromycota	Umbelopsidomycetes	Umbelopsidales	Umbelopsidaceae	*Umbelopsis isabelline* MAFF425103
Mucoromycota	Mucoromycetes	Mucorales	Mucoraceae	*Mucor circinelloides* MAFF425104
Mucoromycota	Mucoromycetes	Mucorales	Rhizopodaceae	*Rhizopus arrhizus* MAFF238040
Ascomycota	Saccharomycetes	Saccharomycetales	Saccharomycetaceae	*Saccharomyces cerevisiae* BY4741
Ascomycota	Sordariomycetes	Glomerellales	Glomerellaceae	*Colletotrichum orbiculare* 104-T MAFF240422
Basidiomycota	Agaricomycetes	Boletales	Suillaceae	Suillus granulatus MAFF435159
Basidiomycota	Ustilaginomycetes	Ustilaginales	Ustilaginaceae	*Ustilago maydis* MAFF511456
Tracheophyta	Magnoliopsida	Fabales	Fabaceae	*Trifolium repens* (white clover)
Tracheophyta	Liliopsida	Poales	Poaceae	*Zea mays* (maize)

### Plant materials

*Lolium multiflorum* roots were sampled in June 2024 in the field (35°54′23.4″N 139°41′11.7″E) of Arai Helmet Ltd., Omiya, Saitama, Japan. Collected roots were removed from the soil on site easily, transferred to the laboratory within 30 min while maintaining humidity. Attached soil was carefully removed with tap water. Care was taken not to damage the roots. The roots were cut into 1 cm lengths with scissors and immersed in 50% ethanol.

### Trypan blue staining of AMF

Ethanol was replaced with 10% KOH, and the roots were heated in a water bath set at 95°C for a period of 10 min. Subsequently, 10% KOH was replaced with 2% HCl and left for a further 10 min at room temperature in order to acidify the interior of the roots. Roots were then treated with a solution of trypan blue (0.05% (w/v) trypan blue, lactic acid) and heated in a water bath at 95°C for 10 min. The roots were then rinsed with deionized water in order to remove any residual trypan blue, after which they were immersed in a lactoglycerol solution (comprising 80% (v/v) lactic acid, 10% (v/v) glycerol, and 10% (v/v) deionized water).

### Sudan IV staining of lipid droplets

The cut roots soaked in 50% ethanol were transferred to 2 ml tubes. After removing the ethanol from the tube, 500 µl of Sudan IV solution (in 70% ethanol) was added and heated in a heating block at 85°C for 10 min.

### PCR and sequencing of fungal rRNA genes in vesicle and root fragment

**Figure 1:**
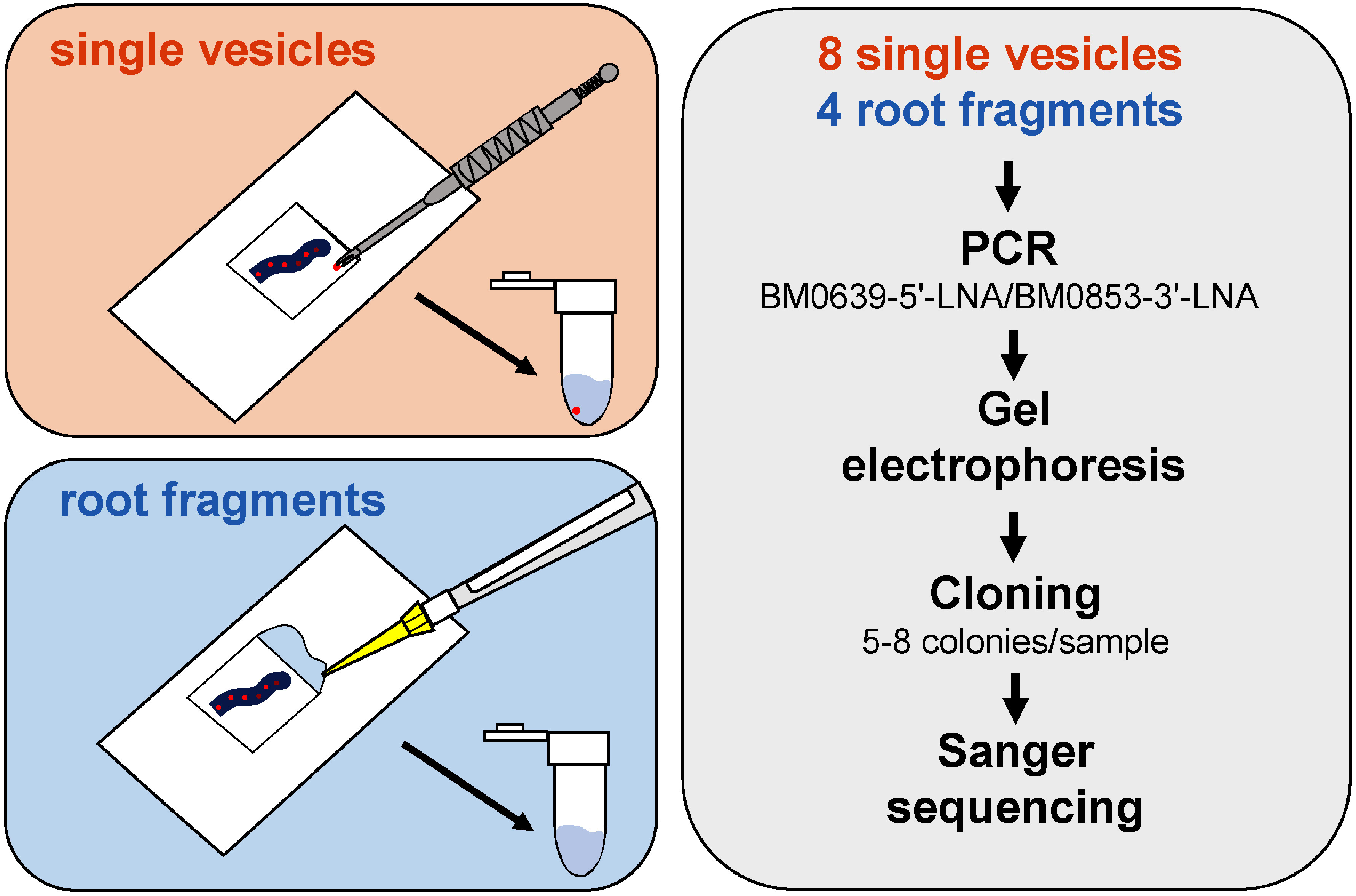
Figure 1. Schematic diagram of mycorrhizal rRNA gene sequencing in vesicles and root fragments. First, roots were cut into approximately 1 cm pieces and stained with Sudan IV. Using a stereomicroscope, root fragments containing lipid-filled vesicles were selected. Top left: lipid-filled vesicles were hollowed out with a sharpened needle tip and transferred to a PCR tube. Bottom left: root fragments (2 mm in length) containing vesicles were placed on a coverslip, 12 µl of Tris-EDTA (pH 8.0) was added, and a smaller coverslip was placed. The sample was crushed and the DNA-containing liquid was collected with a micropipette and 1 µl was transferred to a PCR tube. Mycorrhizal rRNA genes were amplified by PCR with a specific primer set (BM0639-5′-LNA/BM0853-3′-LNA), purified after electrophoresis, cloned into a plasmid vector, and sequenced from 5 to 8 colonies per sample.

Analysis of mycorrhizal roots followed the method of Kobae et al. (2017). Briefly, for single vesicle analysis, a single root fragment (2 mm) containing Sudan IV-positive vesicles was dissected with micro-scissors under a stereomicroscope and placed in 2 µl TE buffer on a square (15×15 mm^2^) of parafilm (Figure 1). A single vesicle was then picked up under the stereomicroscope using a micro-sampling needle (Kajiyama et al. 2015) and transferred directly (with 1 µl TE buffer) into a PCR tube as template for PCR. For root fragment analysis, a single root fragment (2 mm) containing Sudan IV-positive vesicles or -intraradical hyphae was dissected with micro-scissors under a stereomicroscope and placed in 12 µl of TE buffer on a rectangular (24×50 mm^2^) coverslip (Figure 1). The sample was covered with a smaller coverslip (18×18 mm^2^) with caution to avoid the formation of air bubbles between the coverslips. The samples were squashed via pressing with the eraser of a PILOT FRIXION erasable pen (PILOT, Tokyo, Japan). Approximately 5 µl of sample solution that leaked from the side of the upper coverslip were recovered, 1 µl of which was used as the PCR template. KOD One PCR Master Mix (Toyobo) was used for PCR with BM0639-5′-LNA/BM0853-3′-LNA primers. PCR products were separated by gel electrophoresis, excised, and purified for direct sequencing or cloning. For DNA cloning, the PCR products were cloned into pCR®Blunt II-TOPO vector (Thermo Fisher Scientific). The insert sequence was read from M13-Fw on the vector. Sanger sequencing was performed by eurofins Genomics Co. (Tokyo, Japan).

### Phylogenetic analysis

Nucleic acid sequences were aligned using MAFFT v7.475, followed by manual corrections. Phylogenetic trees were constructed using MEGA 11 (Kumar et al. 2018), based on the maximum likelihood method and the Tamura–Nei model (Tamura and Nei 1993). Bootstrap values were calculated based on 100 replicates. The resulting phylogenetic trees were further edited and visualized using iTOL v7 (Letunic and Bork 2024) and Adobe Illustrator 2025.

## Results and discussion

### Design of basal Mucoromycota-specific PCR primers

To detect Mucoromycota fungi colonizing plant roots, we designed taxon-specific primers, BM0639-5′-LNA and BM0853-3′-LNA (Table 1), specifically targeting the 18S rRNA gene of Mucoromycota fungi while avoiding amplification of plant DNA, based on a comparison of 18S rRNA gene sequences from diverse fungal and plant species (Supplementary Figure S1). The sequences of our newly designed primers are very similar to the AM-Sal-F/AMDGR primers, which amplify G-AMF and M-AMF 18S rRNA genes, developed by Seeliger et al. (2024). The primer set BM0639-5′-LNA/BM0853-3′-LNA spans the V4 region of the 18S rRNA gene, producing an amplification product of approximately 260 bp amplicons suitable for sequencing using Illumina MiSeq technology. Although we did not deal with Miseq data in this study, such an analysis is possible in the future using the same primers.

The BM0639-5′-LNA primer is six bases longer at the 5′ end than AM-Sal-F (5′-AAGCTCGTAGTTGAATTT-3′). Additionally, the third thymine (T) from the 3′ end, which mismatches with plant sequences, is replaced with a locked nucleic acid (LNA) base, resulting in a higher T_m_. Sequence comparisons with diverse fungal taxa demonstrate that this primer can effectively bind to most species of Mucoromycota, including Glomeromycetes, Mortierellomycetes, Endogonomycetes, and Umbelopsidomycetes, while excluding Mucoromycetes (Supplementary Figure S1A). However, some species within the families Paraglomeraceae, Diversisporaceae, and Entrophosporaceae of the class Glomeromycetes show a single base mismatch at the third position from the 3′ end of the BM0639-5′-LNA. Similarly, a few species from Mortierellomycetes and Endogonomycetes also exhibit a single base mismatch at the same position. In the case of Ascomycota and Basidiomycota, the mismatches generally range from one to two bases. For plant sequences, the primer has two to three base mismatches.

The BM0853-3′-LNA primer is designed to be two bases shorter at the 5′ end and three bases longer at the 3′ end compared to AMDGR (5′-CCCAACTATCCCTATTAATCAT-3′). To enhance specificity, the sixth adenine (A) from the 5′ end, which mismatches with plant sequences, was replaced with a LNA base (Supplementary Figure S1B). This primer also has two to three base mismatches with the plant sequences. Additionally, a mixed base (adenine or cytosine, represented as M) was introduced at the ninth position from the 3′ end to cover Umbelopsidomycetes sequences. This design allows the primer to effectively bind to most sequences from Glomeromycetes, Mortierellomycetes, Endogonomycetes, and Umbelopsidomycetes. For Mucoromycetes, there are two to five mismatches. Ascomycota and Basidiomycota show a few mismatches. However, the primer exhibits complete matches with some sequences from Agaricales and Russulales.

### Primer specificity

**Figure 2:**
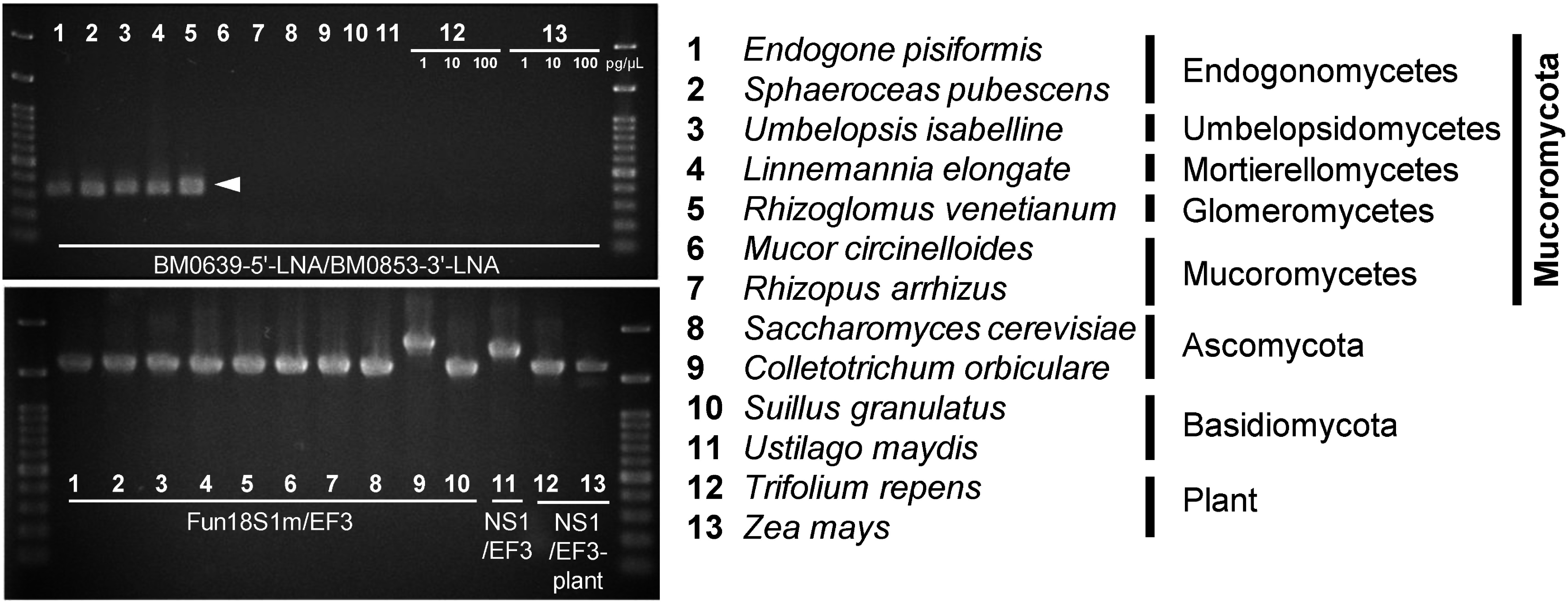
Figure 2. Primer specificity. The basal Mucoromycota-specific primer set BM0639-5′-LNA/BM0853-3′-LNA, linked with Illumina MiSeq adaptors (Table 1), was utilized to amplify diverse fungal and plant 18S rRNA genes cloned into the pZErO-2 plasmid (upper panel). For fungal gene amplification, plasmid DNA at a concentration of 1 pg µl^−1^ was used as the template. For plant gene amplification, plasmid DNA at concentrations of 1, 10, and 100 pg µl^−1^ was added to PCR mixtures. A 100 bp DNA ladder was used as the molecular markers. Approximately 320 bp amplicons (arrowhead) were successfully obtained from *E. pisiformis*, *S. pubescens*, *U. isabellina*, *L. elongata*, and *R. venetianum*. Positive controls for PCR amplification were performed using universal primer sets (lower panel). Plasmid DNA at a concentration of 1 pg µl^−1^ was used for PCR.

The BM0639-5′-LNA/BM0853-3′-LNA primer set was tested by PCR amplification using the cloned 18S rRNA sequences. At an annealing temperature of 60°C, successful amplification was observed for the target species *Endogone pisiformis*, *S. pubescens*, *Umbelopsis isabelline*, *Linnemannia elongata*, and *Thiazoliums venetianum* (Figure 2; Table 2). No amplification occurred with non-target species, and no amplification occurred even in the presence of high concentrations of plant DNA. These results confirm that the BM0639-5′-LNA/BM0853-3′-LNA primer set specifically amplifies the 18S rRNA genes of most Mucoromycota, excluding Mucoromycetes.

### Ribosomal RNA genes in single vesicles

**Figure 3:**
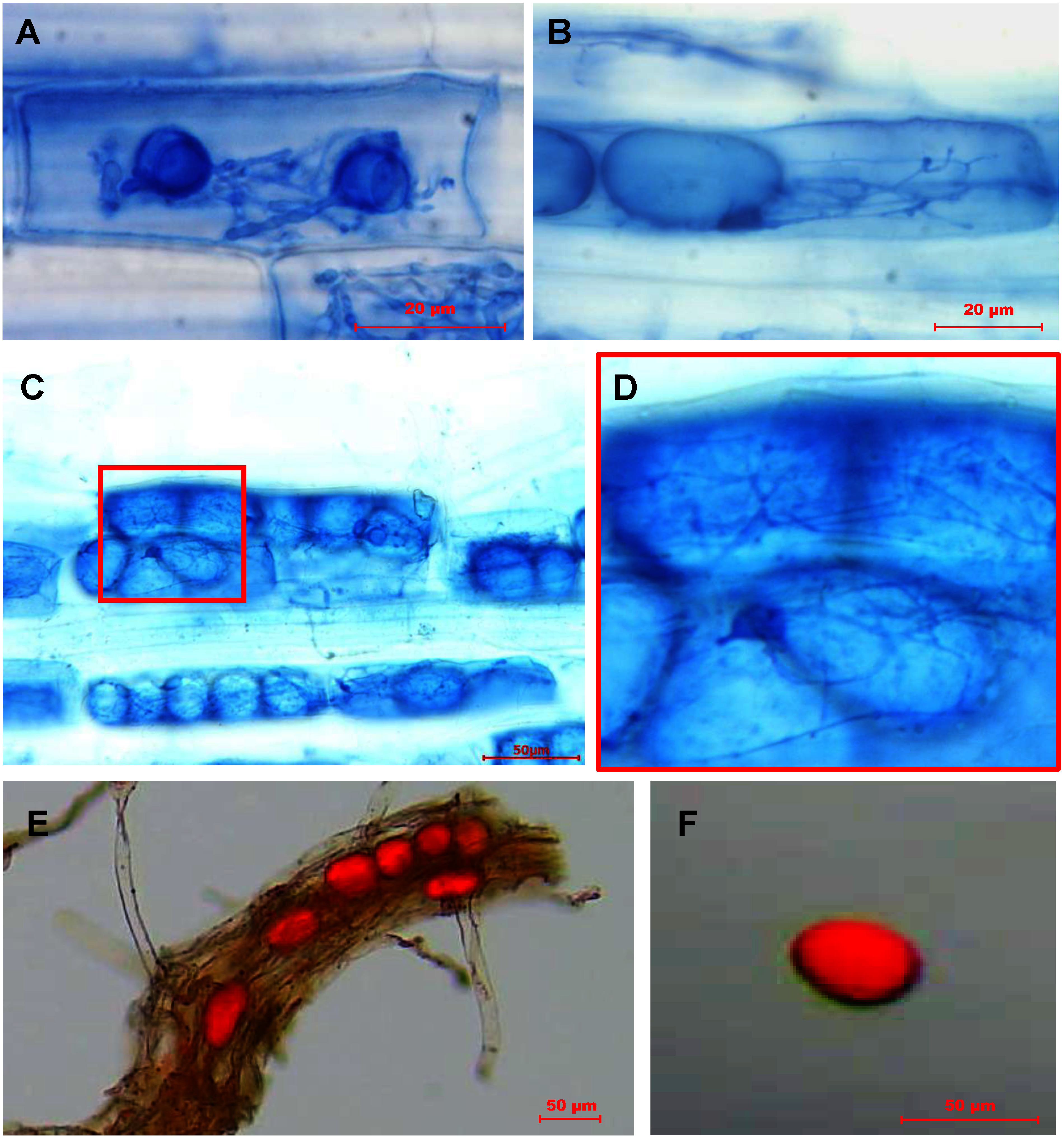
Figure 3. Vesicles formed in the roots of *Lolium multiflorum*. A–D, Roots stained with trypan blue. Vesicles re formed intracellularly. D, A magnified image of the red boxed area in C. Numerous thin hyphae are seen around the vesicles. E and F, Sudan IV staining of the root. E, Lipids in the vesicle are stained red. F, A vesicle hollowed out with a sharpened needle tip.

*Lolium multiflorum* plants sampled in June 2024 were at the stage of seed maturation, and trypan blue staining of the roots revealed numerous vesicles. These vesicles were not formed in the intercellular spaces, as are the usual G-AMF vesicles, but within the cells (Figure 3). While vesicle formation in G-AMF often results in the enlargement of hyphal terminals in the intercellular space, this vesicle formation is accompanied by intracellular, but less than arbuscule, hyphal branching (Figure 3A, B). In addition, many fine mycelia were adhering to the periphery of the vesicles (Figure 3C, D). This morphology is similar to the “lumps” of M-AMF observed in parenchyma cells of liverwort mycothalli (Carafa et al. 2003; Duckett et al. 2006; Field et al. 2015). Detailed morphological observations of mycothalli predict that these lumps collapse within a short time and are taken up by plant cells to become a source of carbon and nitrogen for the plant (Howard et al. 2022). However, many of the vesicles formed in the *L. multiflorum* roots developed large enough to occupy much of the cell volume (Figure 3C, D), and there was no morphological evidence that they collapse in a short period of time. These vesicles were also observed in senescent roots and exhibit the static characteristics of long-term nutrient storage rather than the dynamic nutrient exchange. It is unclear whether the characteristic morphology of the vesicles observed in *L. multiflorum* root is due to the specific growth stage of *L. multiflorum*, specific to this plant, specific to the AMFs in this field, or a universal phenomenon of M-AMF/G-AMF colonization, we need to examine more different samples from different fields. In particular, the time course of morphological changes needs to be studied in detail, which requires new analytical methods at the cellular level to distinguish whether the structure is G-AMF or M-AMF.

These vesicles, like the G-AMF vesicles, accumulate large amounts of lipid droplets, which was also evident by trypan blue staining. Since trypan blue-stained roots cannot be used for their genetic analysis because their DNA was damaged by the KOH treatment, the lipid droplets were stained with Sudan IV (70% ethanol as solvent) to visualize the vesicles in the roots (Figure 3E). To examine the AMF species in a single vesicle, vesicles were hollowed out with a sharpened needle tip. (Kajiyama et al. 2015) (Figure 1; Figure 3F). Using the isolated vesicles as templates, PCR was performed with the BM0639-5′-LNA/BM0853-3′-LNA primer set, and directly sequenced. Direct sequencing clearly showed that the single vesicle contained multiple rRNA gene sequences intermingled (i.e., multilayered electropherogram suggesting a mixture of several different gene sequences) (Kobae et al. 2017). This is consistent with recent reports that the AMF genome has multiple heterogeneous rRNA genes (Maeda et al. 2018; Sperschneider et al. 2023; Yildirir et al. 2022). Therefore, we cloned the purified PCR products into plasmid vectors and sequenced 5–8 colonies from each vesicle. The PCR products of the root fragments were also cloned in the same way and the sequences of 5–8 colonies were determined

**Figure 4:**
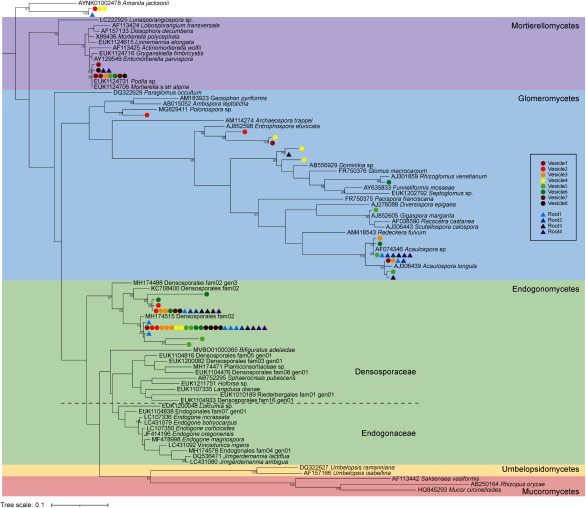
Figure 4. A maximum likelihood tree based on partial 18S rDNA sequences obtained from isolated vesicles (vesicle1–vesicle8) and single root segments of *Lolium multiflorum* containing Sudan IV-positive vesicles or -intraradical hyphae (root1–root4). These sequences were amplified using the primer set BM0639-5′-LNA/BM0853-3′-LNA and cloned into pCR Blunt II-TOPO. Seven to eight clones per sample were sequenced and aligned with fungal reference sequences from publicly available databases. The tree is rooted with *Amanita jacksonii*. Bootstrap values (from 100 replicates) greater than 50% are shown below the branches. Sequences of the samples are represented as colored circles.

Phylogenetic analysis was performed using the obtained sequences and reference rRNA gene sequences of Mucoromycota (Endogonomycetes, Umbelopsidomycetes, Mortierellomycetes, Glomeromycetes, and Mucoromycetes). Out of a total of 84 sequences tested, none were classified as Mucoromycetes, Ascomycota, and plants, supporting the specificity of this primer set (Figure 4). However, some sequences were closest to the basidiomycete *Amantia jacksonii* among the reference sequences. BLAST searches of these sequences showed the highest similarity to *Malassezia*, an endemic fungus of human skin. This amplified sequence could be a contaminant during sample preparation and it is likely that this primer set amplifies parts of the basidiomycete.

The majority of the cloned rRNA gene sequences were classified as Endogonomycetes, Glomeromycetes, and Mortierellomycetes. Surprisingly, not only the cloned rRNA gene sequences from the 2 mm root fragments, where multiple species can co-colonize, but also the rRNA gene sequences isolated from a single vesicle were classified into clades spanning multiple genera (Figure 4). This suggests that the isolated vesicles contain multiple fungal species. Although it is known that multiple AMF species can be simultaneously detected even a 1 cm root (van Tuinen et al. 1998), to our knowledge there are no reports of the co-occurrence of mycorrhizal fungi of different genera in a single vesicle. Nevertheless, it is possible that the thin mycelium adhering to the vesicle was a different species from the vesicle. Notably, numerous studies have recognized that G-AMF and M-AMF are always co-occurring in roots, albeit in different proportions (Albornoz et al. 2021; Orchard et al. 2017a; Walker et al. 2018; Yamamoto et al. 2019). Quantitative discussion of the G-AMF/M-AMF is still difficult at present, as the copy number of rRNA genes in the genome of M-AMF and the number of nuclei in the mycelium are unknown. The biological and ecological reasons for such a high frequency of co-occurrence need to be further investigated. The vesicles studied in this research may be qualitatively different from the G-AMF vesicles observed in many previous studies. These vesicles were formed within cells and were observed in the roots of the reproductive stage of *L. multiflorum*. Unfortunately, we do not have enough information here to discuss the actual nature of these vesicles. It will be necessary to investigate in the future whether similar modes of colonization and heterologous rRNA genes are found in the vesicles of roots from other fields and other plants.

## Conclusion

Recently, M-AMF has attracted much interest because of its ability to provide both nitrogen and phosphate to plants. However, there is still very little information on how many species of M-AMF are present in the roots of plants in the field. In addition, little is known about which species have which morphological characteristics and physiological roles. Because the majority of M-AMF cannot be cultured, it is not easy to match the identity of M-AMF with its characteristics. Identification of species at the tissue/cellular level based on detectable unique features is necessary for future research.

Genetic information is useful as a method of identification; it has been pointed out that species classification at the genomic level is necessary due to the heterogeneity of the rRNA genes possessed by AMF (Corradi et al. 2025). On the other hand, it is also necessary to distinguish between G-AMF and M-AMF and to analyze their lifestyle by simpler methods. This requires specific primers that can efficiently amplify both G-AMF and M-AMF simultaneously, have sufficient classification resolution to elucidate novel functions and properties, and exclude fungi unrelated to the mycorrhizal symbiosis. The primers designed in this study did not amplify Mucoromycetes, which are not known to form mycorrhizae. In addition, the Mortierellomycetes were shown to be phylogenetically close to the mycorrhizal fungi Endogonomycetes and Glomeromycetes based on a recent genome-level phylogenetic analysis (Rosling et al. 2024). However, it is not clear whether Mortierellomycetes form arbuscules or how they exchange nutrients with plants. Our approach appears to be valid for large-scale analyzes using whole roots from many different conditions, as well as for microscale analyzes at the cellular level. Of particular note is that the season and environment in which M-AMF is observed is likely to be different from that of G-AMF. To gain a deeper insight into the lifestyle and nature of M-AMF, it is necessary to study the mycorrhiza of a wide variety of plants in different ecosystems.

## Abbreviations

AMF: arbuscular mycorrhizal fungi
FRE: fine root endophytes
LNA: locked nucleic acid
18S rRNA: 18S small subunit of ribosomal RNA

## Accession numbers

The nucleotide sequences have been submitted to the DNA Data Bank of Japan (https://www.ddbj.nig.ac.jp/index-e.html) under the accession numbers LC863045–LC863137.

## Description of supplementary files

Supplementary Figure S1. Alignment of plant and fungal 18S rRNA sequences with basal Mucoromycota-specific primers.
