# Supplementary material for: Various types of mycorrhizal fungi sequences detected in single intracellular vesicles
Source: Plant Biotechnol (Tokyo). 2025 Sep 25;42(3):299–307. doi: 10.5511/plantbiotechnology.25.0228a (PMC12573499; doi:10.5511/plantbiotechnology.25.0228a)
Supplement: Supplementary Data [file plantbiotechnology-42-3-25.0228a_s001.pdf]

A

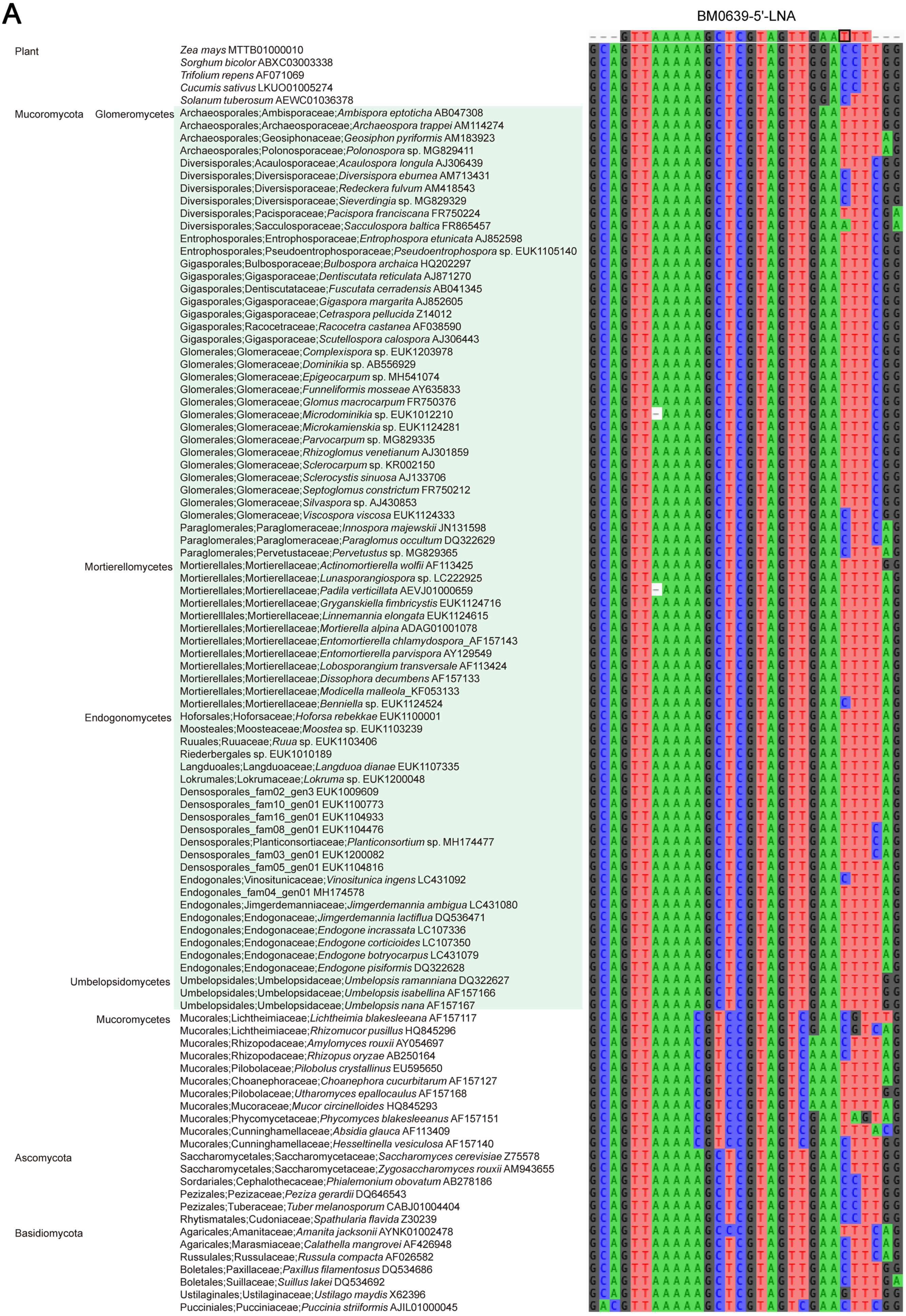

BM0639-5'-LNA
Plant
Zea mays MTTB01000010
Sorghum bicolor ABXC03003338
Trifolium repens AF071069
Cucumis sativus LKUO01005274
Solanum tuberosum AEWC01036378
Mucoromycota
Glomeromycetes
Archaeosporales;Ambisporaceae;Ambispora eptoticha AB047308
Archaeosporales;Archaeosporaceae;Archaeospora trappei AM114274
Archaeosporales;Geosiphonaceae;Geosiphon pyriformis AM183923
Archaeosporales;Polonosporaceae;Polonospora sp. MG829411
Diversisporales;Acaulosporaceae;Acaulospora longula AJ306439
Diversisporales;Diversisporaceae;Diversispora eburnea AM713431
Diversisporales;Diversisporaceae;Redeckera fulvum AM418543
Diversisporales;Diversisporaceae;Sieverdingia sp. MG829329
Diversisporales;Pacisporaceae;Pacispora franciscana FR750224
Diversisporales;Sacculosporaceae;Sacculospora baltica FR865457
Entrophosporales;Entrophosporaceae;Entrophospora etunicata AJ852598
Entrophosporales;Pseudoentrophosporaceae;Pseudoentrophospora sp. EUK1105140
Gigasporales;Bulbosporaceae;Bulbospora archaica HQ202297
Gigasporales;Gigasporaceae;Dentiscutata reticulata AJ871270
Gigasporales;Dentiscutataceae;Fuscutata cerradensis AB041345
Gigasporales;Gigasporaceae;Gigaspora margarita AJ852605
Gigasporales;Gigasporaceae;Cetraspora pellucida Z14012
Gigasporales;Racocetraceae;Racocetra castanea AF038590
Gigasporales;Gigasporaceae;Scutellospora calospora AJ306443
Glomerales;Glomeraceae;Complexispora sp. EUK1203978
Glomerales;Glomeraceae;Dominikia sp. AB556929
Glomerales;Glomeraceae;Epigeocarpum sp. MH541074
Glomerales;Glomeraceae;Funneliformis mosseae AY635833
Glomerales;Glomeraceae;Glomus macrocarpum FR750376
Glomerales;Glomeraceae;Microdominikia sp. EUK1012210
Glomerales;Glomeraceae;Microkamienskia sp. EUK1124281
Glomerales;Glomeraceae;Parvocarpum sp. MG829335
Glomerales;Glomeraceae;Rhizoglomus venetianum AJ301859
Glomerales;Glomeraceae;Sclerocarpum sp. KR002150
Glomerales;Glomeraceae;Sclerocystis sinuosa AJ133706
Glomerales;Glomeraceae;Septoglomus constrictum FR750212
Glomerales;Glomeraceae;Silvaspora sp. AJ430853
Glomerales;Glomeraceae;Viscospora viscosa EUK1124333
Paraglomerales;Paraglomeraceae;Innospora majewskii JN131598
Paraglomerales;Paraglomeraceae;Paraglomus occultum DQ322629
Paraglomerales;Pervetustaceae;Pervetustus sp. MG829365
Mortierellomycetes
Mortierellales;Mortierellaceae;Actinomortierella wolfii AF113425
Mortierellales;Mortierellaceae;Lunasporangiospora sp. LC222925
Mortierellales;Mortierellaceae;Padila verticillata AEVJ01000659
Mortierellales;Mortierellaceae;Gryganskiella fimbricystis EUK1124716
Mortierelllales;Mortierellaceae;Linnemannia elongata EUK1124615
Mortierellales;Mortierellaceae;Mortierella alpina ADAG01001078
Mortierellales;Mortierellaceae;Entomortierella chlamydospora_AF157143
Mortierellales;Mortierellaceae;Entomortierella parvispora AY129549
Mortierellales;Mortierellaceae;Lobosporangium transversale AF113424
Mortierellales;Mortierellaceae;Dissophora decumbens AF157133
Mortierellales;Mortierellaceae;Modicella malleola_KF053133
Mortierellales;Mortierellaceae;Benniella sp. EUK1124524
Endogonomycetes
Hoforsales;Hoforsaceae;Hoforsa rebekkae EUK1100001
Moosteales;Moosteaceae;Moostea sp. EUK1103239
Ruuales;Ruuaceae;Ruua sp. EUK1103406
Riederbergales sp. EUK1010189
Langduoales;Langduoaceae;Langduoa dianae EUK1107335
Lokrumales;Lokrumaceae;Lokruma sp. EUK1200048
Densosporales_fam02_gen3 EUK1009609
Densosporales_fam10_gen01 EUK1100773
Densosporales_fam16_gen01 EUK1104933
Densosporales_fam08_gen01 EUK1104476
Densosporales;Planticonsortiaceae;Planticonsortium sp. MH174477
Densosporales_fam03_gen01 EUK1200082
Densosporales_fam05_gen01 EUK1104816
Endogonales;Vinositunicaceae;Vinositunica ingens LC431092
Endogonales_fam04_gen01 MH174578
Endogonales;Jimgerdemanniaceae;Jimgerdemannia ambigua LC431080
Endogonales;Endogonaceae;Jimgerdemannia lactiflua DQ536471
Endogonales;Endogonaceae;Endogone incrassata LC107336
Endogonales;Endogonaceae;Endogone corticioides LC107350
Endogonales;Endogonaceae;Endogone botryocarpus LC431079
Endogonales;Endogonaceae;Endogone pisiformis DQ322628
Umbelopsidomycetes
Umbelopsidales;Umbelopsidaceae;Umbelopsis ramanniana DQ322627
Umbelopsidales;Umbelopsidaceae;Umbelopsis isabellina AF157166
Umbelopsidales;Umbelopsidaceae;Umbelopsis nana AF157167
Mucoromycetes
Mucorales;Lichtheimiaceae;Lichtheimia blakesleeana AF157117
Mucorales;Lichtheimiaceae;Rhizomucor pusillus HQ845296
Mucorales;Rhizopodaceae;Amylomyces rouxii AY054697
Mucorales;Rhizopodaceae;Rhizopus oryzae AB250164
Mucorales;Pilobolaceae;Pilobolus crystallinus EU595650
Mucorales;Choanephoraceae;Choanephora cucurbitarum AF157127
Mucorales;Pilobolaceae;Utharomyces epallocaulus AF157168
Mucorales;Mucoraceae;Mucor circinelloides HQ845293
Mucorales;Phycomycetaceae;Phycomyces blakesleeanus AF157151
Mucorales;Cunninghamellaceae;Absidia glauca AF113409
Mucorales;Cunninghamellaceae;Hesseltinella vesiculosa AF157140
Ascomycota
Saccharomycetales;Saccharomycetaceae;Saccharomyces cerevisiae Z75578
Saccharomycetales;Saccharomycetaceae;Zygosaccharomyces rouxii AM943655
Sordariales;Cephalothecaceae;Phialemonium obovatum AB278186
Pezizales;Pezizaceae;Peziza gerardii DQ646543
Pezizales;Tuberaceae;Tuber melanosporum CABJ01004404
Rhytismatales;Cudoniaceae;Spathularia flavida Z30239
Basidiomycota
Agaricales;Amanitaceae;Amanita jacksonii AYNK01002478
Agaricales;Marasmiaceae;Calathella mangrovei AF426948
Russulales;Russulaceae;Russula compacta AF026582
Boletales;Paxillaceae;Paxillus filamentosus DQ534686
Boletales;Suillaceae;Suillus lakei DQ534692
Ustilaginales;Ustilaginaceae;Ustilago maydis X62396
Pucciniales;Pucciniaceae;Puccinia striiformis AJIL01000045

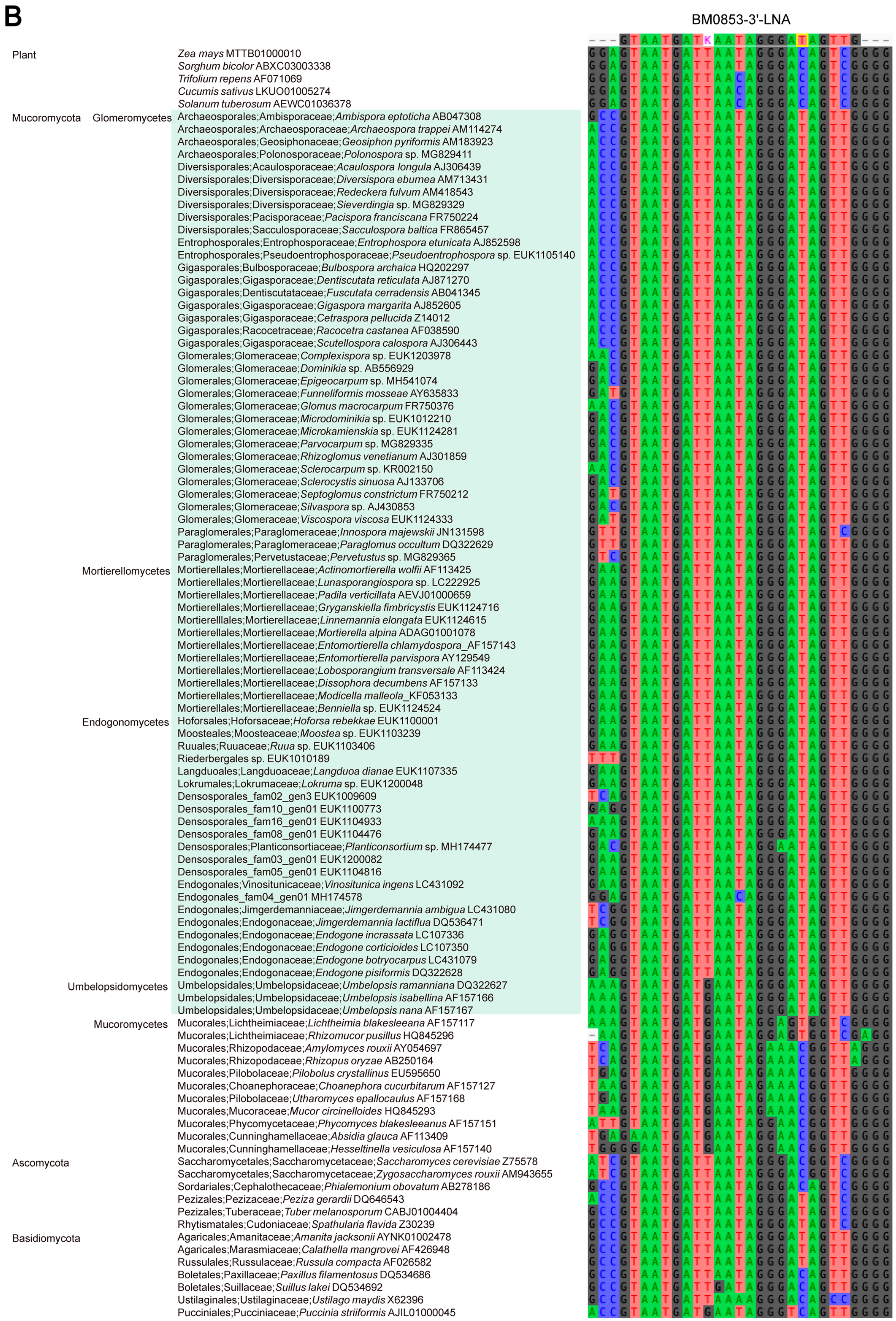

Supplementary Figure S1. Alignment of plant and fungal 18S rRNA sequences with basal Mucoromycota-specific primers. (A) The forward primer BM0639-5'-LNA. The primer binds to positions 616 to 639 nt, referenced to the Saccharomyces cerevisiae 18S rRNA gene sequence (accession number Z75578). Locked Nucleic Acid (LNA) is indicated a square. (B) The reverse primer BM0853-3'-LNA. The primer binds to positions 875 to 853 nt, referenced to the Saccharomyces cerevisiae 18S rRNA gene sequence (accession number Z75578). Locked Nucleic Acid (LNA) is indicated a square.
